# Case Report: Neuromyelitis optica spectrum disorder associated with anti-argonaute antibodies presenting with subacute combined degeneration-like features

**DOI:** 10.3389/fimmu.2026.1742459

**Published:** 2026-04-10

**Authors:** Fangyuan Yi, Xunyu Yang, Ruiping Wang

**Affiliations:** 1 Department of Neurology, Jin Qiu Hospital of Liaoning Province (Geriatric Hospital of Liaoning Province), Shenyang, Liaoning, China; 2 College of Chemistry and Chemical Engineering, Shenyang Normal University, Shenyang, Liaoning, China

**Keywords:** aquaporin-4 antibody, argonaute antibody, longitudinally extensive myelitis, multimodal assessments, neuromyelitis optica spectrum disorder

## Abstract

Aquaporin-4 antibodies (AQP4-Abs) are a key diagnostic biomarker of neuromyelitis optica spectrum disorder (NMOSD), and anti-Argonaute antibodies (AGO-Abs) have recently been reported in a range of autoimmune neurological conditions, although their clinical significance remains undetermined. In this report, we describe a 36-year-old woman who initially presented with sensory disturbances and mild vitamin B12 deficiency who was initially diagnosed with subacute combined degeneration. Vitamin supplementation partially improved her symptoms but her condition subsequently deteriorated, and she developed paraparesis, ascending sensory loss, and urinary incontinence. Magnetic resonance imaging revealed longitudinally extensive transverse myelitis with gadolinium enhancement. Serum and cerebrospinal fluid analyses were positive for AQP4-Abs and AGO-Abs, supporting the diagnosis of NMOSD with concomitant presence of AGO antibodies. Electrophysiological studies showed asymmetric axonal sensorimotor polyneuropathy, suggesting peripheral nervous system involvement contributing to early diagnostic uncertainty. The patient received immunotherapy including high-dose corticosteroids, intravenous immunoglobulin, and plasma exchange, after which marked neurological improvement was observed. Longitudinal multimodal assessments incorporating antibody titers, expanded disability status scale scores, somatosensory-evoked potentials, and neuroimaging were performed during follow-up. This case highlights the heterogeneous clinical presentation of NMOSD, the potential coexistence of AGO antibodies, and the possibility that serological changes may not parallel clinical recovery. Comprehensive antibody evaluation and multimodal monitoring may assist clinical decision-making in atypical neuroimmunological presentations.

## 1 Introduction

Neuromyelitis optica spectrum disorder (NMOSD) is a rare, relapsing autoimmune disorder that predominantly affects women, with a female-to-male ratio of approximately 8-9:1, and often occurs during pregnancy or the postpartum period (1). Although the majority (<75%) of patients have pathogenic aquaporin-4 antibodies (AQP4-Abs), seronegative cases have also been reported (2). Argonaute antibodies (AGO-abs) are a novel class of autoantibodies targeting argonaute proteins, which are core effectors in RNA-induced silencing pathways, and they have been identified in systemic autoimmune diseases such as systemic lupus erythematosus (SLE), Sjögren’s syndrome, and dermatomyositis (3, 4). Emerging evidence suggests there are neurological associations, including autoimmune encephalitis and immune-mediated peripheral neuropathies (5, 6). Recent observations have further expanded this spectrum, with anti-AGO-Abs reported in Epstein-Barr virus encephalitis, indicating that AGO-Ab positivity may also occur in infection-related neuroinflammatory conditions (7).

Although the coexistence of NMOSD and AGO-Abs positivity has been reported previously, the clinical significance of this association remains incompletely understood. Here, we present a patient with AQP4-positive NMOSD accompanied by AGO-Abs positivity who had peripheral nervous system involvement. This case illustrates how rather than representing a distinct syndrome, this case illustrates how coexisting autoimmune antibody profiles and metabolic abnormalities may complicate early diagnosis and therapeutic decision-making. Our findings further contribute to the emerging spectrum of antibody coexistence in NMOSD and highlight the importance of comprehensive immunological evaluation and therapeutic strategies in atypical neuroimmunological presentations.

## 2 Case report

**Figure 1:**
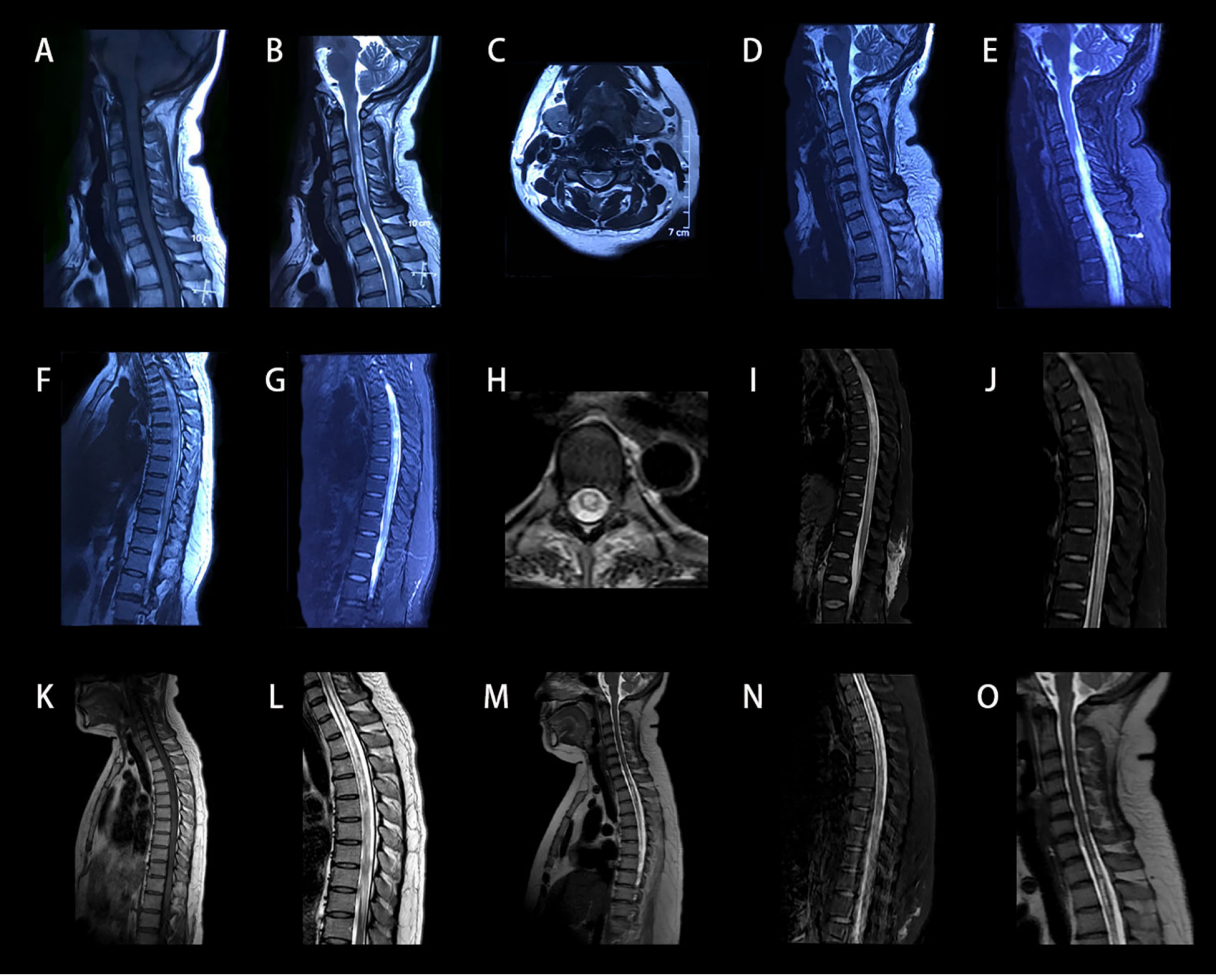
Cervical MRI performed during acute phase of disease. **(A–C)** Sagittal and axial T1 or T2-weighted images of cervical MRI scans at first-onset show posterior cord lesions along the C2-C6 levels and upper thoracic segments, without enhancement. **(D–J)** Cervical MRI shows longitudinally extensive abnormal spinal cord signal intensity with long T1-weighted **(D, F)** and long T2-weighted **(E, G)** at C2-T11 levels with swelling. The Gd-enhanced MRI showed significant enhancement of cervical and thoracic spinal cord lesions **(H–J)**. **(K, L)** Following combined IVMP and IVIG therapy, MRI demonstrates reduction of spinal cord swelling with decreased hyperintense signal intensity extending from C6 to T10 levels. The Gd-enhanced MRI showed no significant enhancement of spinal lesions, but syringomyelia were present at C7-T2. **(M–O)** Follow-up MRI following first-line therapy demonstrates multi-focal patchy hyperintensities spanning T1-T10 on T2, and mild enlargement of syringomyelia compared to prior imaging, extending from C6 to T4. MRI, magnetic resonance imaging; Gd-enhanced, gadolinium enhanced; IVMP, intravenous methylprednisolone; IVIG, intravenous immunoglobulin.

A 36-year-old woman presented with a 4-month history of bilateral hand numbness and weakness, toe paresthesia which developed into progressive lower limb weakness and sensory loss, and 10 days of urinary incontinence. She denied any history of rash, arthralgia, xerostomia, or dry eyes. The symptoms began with bilateral hand numbness and weakness, progressing to loss of grasp, toe numbness, and urinary retention. Spinal magnetic resonance imaging (MRI) demonstrated a longitudinal posterior column lesion at the level of C2-C6 and upper thoracic segments (**Figures 1A–C**).

Laboratory tests revealed serum vitamin B12 was mildly reduced (157 pg/ml, reference range 180–914 pg/ml). Some autoantibodies were mildly elevated, including anti-intrinsic factor antibodies (1.48 Au/ml, normal value: < 1.20 Au/ml), cytoplasmic antinuclear antibodies (ANA, 1:80), antimitochondrial antibodies (AMA, 1:160), and antimitochondrial antibody subtype-M2 (AMA-M2, 180.28 RU/ml; normal value: < 20 RU/ml). Cerebrospinal fluid (CSF) examination was not performed at the time. No clear history of malnutrition, vegetarian diet, alcohol abuse, or other nutritional risk factors was identified. Homocysteine and methylmalonic acid levels were not obtained at the time. Based on the presence of vitamin B12 deficiency, sensory symptoms, urinary dysfunction, and early myelopathic features, a diagnosis of subacute combined degeneration (SCD) was initially made at a local clinic. The patient was treated with a combination of vitamin B1, vitamin B6, mecobalamin, and folic acid. Her symptoms of hand weakness and urinary retention completely resolved after 1 week of treatment. Following 2 months of ongoing treatment, the numbness in her extremities gradually improved until it was limited to the fourth and fifth digits of her left hand.

Approximately four months after the initial symptom onset, and ten days prior to re-admission, she developed heaviness in the left calf and foot drop. Within 24 hours, her symptoms rapidly progressed to left leg weakness, right foot paresthesia, and complete bilateral lower limb paralysis. Sensory disturbances ascended to the T2 dermatome, and she also developed recurrent urinary incontinence. Repeat cervical and thoracic MRI revealed longitudinally extensive lesions extending from C2 to T11 with cord swelling and patchy gadolinium enhancement (**Figures 1D–J**). She was admitted under the provisional diagnosis of demyelinating myelitis. Four years previously, she had a history of right optic neuritis treated with a one-month course of oral corticosteroids, resulting in residual visual impairment. In addition, she had undergone a cesarean section 3 months prior to this admission, delivering a healthy male infant.

On admission, the patient’s vital signs were within normal limits: body temperature of 36.5 °C, pulse rate 70 bpm, respiratory rate 18 bpm, blood pressure 107/75 mmHg, and body mass index of 33.06 kg/m^2^. Cardiopulmonary and abdominal examinations were unremarkable. Neurological examination revealed no cognitive and cranial nerve deficits. The upper limbs had normal strength and coordination, while the lower limbs showed flaccid paraplegia (grade 0 strength) with absent ankle reflexes. Deep tendon reflexes in the upper limbs and knees were normal (2+). Sensory examination revealed severe impairment of superficial and vibration sensation below the T2 level. Finger-to-nose and rapid alternating movement tests were intact in both upper limbs. Lower limb ataxia could not be assessed due to paraplegia. Babinski and Chaddock signs were positive on the right side, and the anal reflex was absent. The Expanded Disability Status Scale (EDSS) score was 8.5 based on the clinical manifestations (wheelchair dependence with severe paraplegia and sensory deficits).

Routine serum, urine, and stool analyses yielded normal findings. Erythrocyte sedimentation rate, liver and renal function tests, electrolyte panels, blood glucose, and thyroid function were all within normal limits. Serum ANA was weakly positive with a cytoplasmic pattern (1:80). Humoral immunity tests showed elevated levels of immunoglobulin A to 4.04 (reference range: 0.7–4 g/L), while the levels of immunoglobulins G, E, M and complement were normal. Tuberculosis, rapid plasma regain, HIV, hepatitis B, and hepatitis C antibodies were negative.

**Figure 2:**
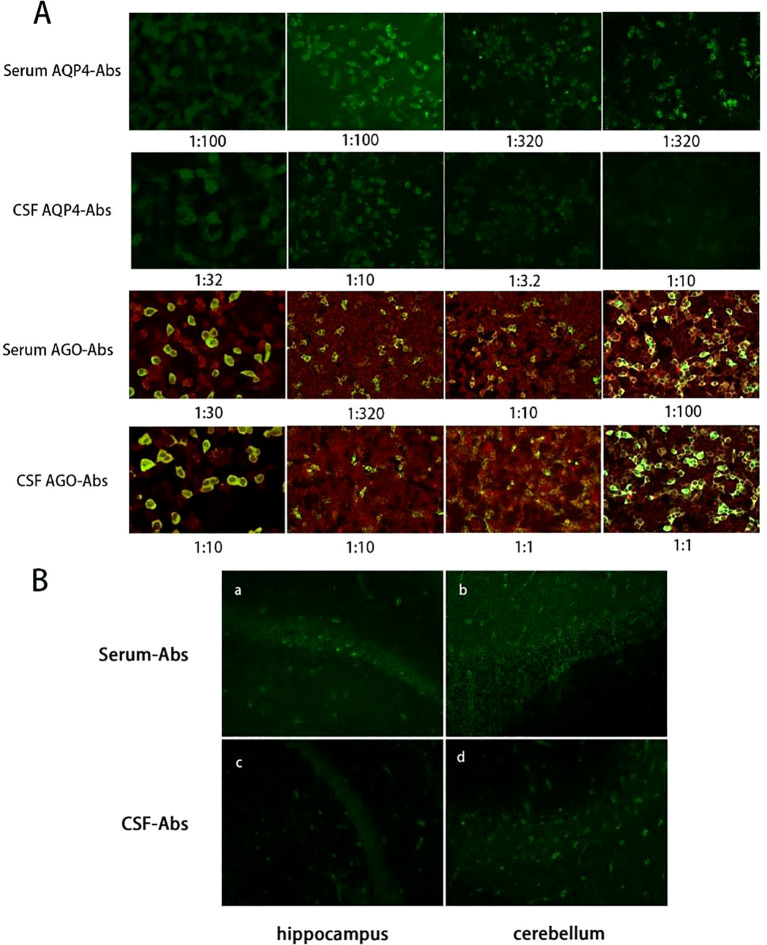
Detection of AQP4-Abs and AGO-Abs by CBA and TBA. **(A)** CBA of AQP4-Abs or AGO-Abs titer levels in serum and CSF at different stages. The first column is of results before treatment, the second column after IVMP and IVIG, and the third column after PE treatment. All antibodies except serum AQP4-Abs decreased significantly after first-line treatment. The fourth column shows antibody titer levels at 9-month follow-up. Although clinical symptoms had markedly improved, serum antibody titers remained elevated, whereas cerebrospinal fluid antibody titers decreased. **(B)** IIF of monkey brain with the patient’s serum **(a, b)** and CSF **(c, d)** shows strong reactivity with the hippocampal stratum pyramidale and cerebellar granule cells. CBA, cell-based assay; TBA, tissue-based assay; AQP4-Abs, aquaporin-4 antibodies; AGO-Abs, argonaute antibodies; CSF, cerebrospinal fluid; IVIG, intravenous immunoglobulin; IVMP, intravenous methylprednisolone; PE, plasma exchange; IIF, indirect immunofluorescence.

The CSF pressure was 180 mmH_2_O. Routine CSF analysis revealed an elevated white blood cell count of 31 × 10^6^/L, predominantly mononuclear cells (94.5%). Biochemical values were normal (glucose, 3.17 mmol/L; chlorine, 128.8 mmol/L; and protein, 0.47 g/L), and interleukin-6 (IL-6, 7.84 pg/mL). The bacterial and fungal cultures, CSF tuberculosis, and acid-fast Bacillus ink staining were negative. Due to limitations imposed by medical insurance, the autoimmune antibody tests were outsourced to a third-party detection institution (Hightrust Diagnostic Co., Ltd., Beijing, China). Cell-based assay (CBA) was performed on serum and CSF samples to detect antibodies associated with central nervous system demyelinating diseases and autoimmune encephalitis. The results showed that both AQP4-Abs and AGO-Abs were positive in serum and CSF, with titer levels shown in **Figure 2A**. Further CSF analysis revealed elevated immunoglobulin G (IgG) levels (57.6 mg/L; normal range 10–30 mg/L). The IgG index was calculated as 0.44 (normal <0.7), and oligoclonal bands (OCB) were type I, indicating no intrathecal IgG synthesis. Furthermore, a tissue-based assay (TBA) of the monkey brain with serum and CSF was performed for autoantibody detection, which showed in strong reactivity with the hippocampal stratum pyramidale and cerebellar granule cells (**Figure 2B**).

The patient was diagnosed with overlapping NMOSD and AGO-Abs syndrome. She received an intravenous pulse therapy regimen of methylprednisolone (1g/day for 3 days, 500 mg/day for 3 days, 250 mg/day for 3 days, and 120 mg/day for 3 days). This regimen was followed by weaning on oral prednisone, initiated at 60 mg/day and reduced by 10 mg per week, in accordance with the Chinese Consensus Guidelines for the Management of NMOSD (8).

**Figure 3:**
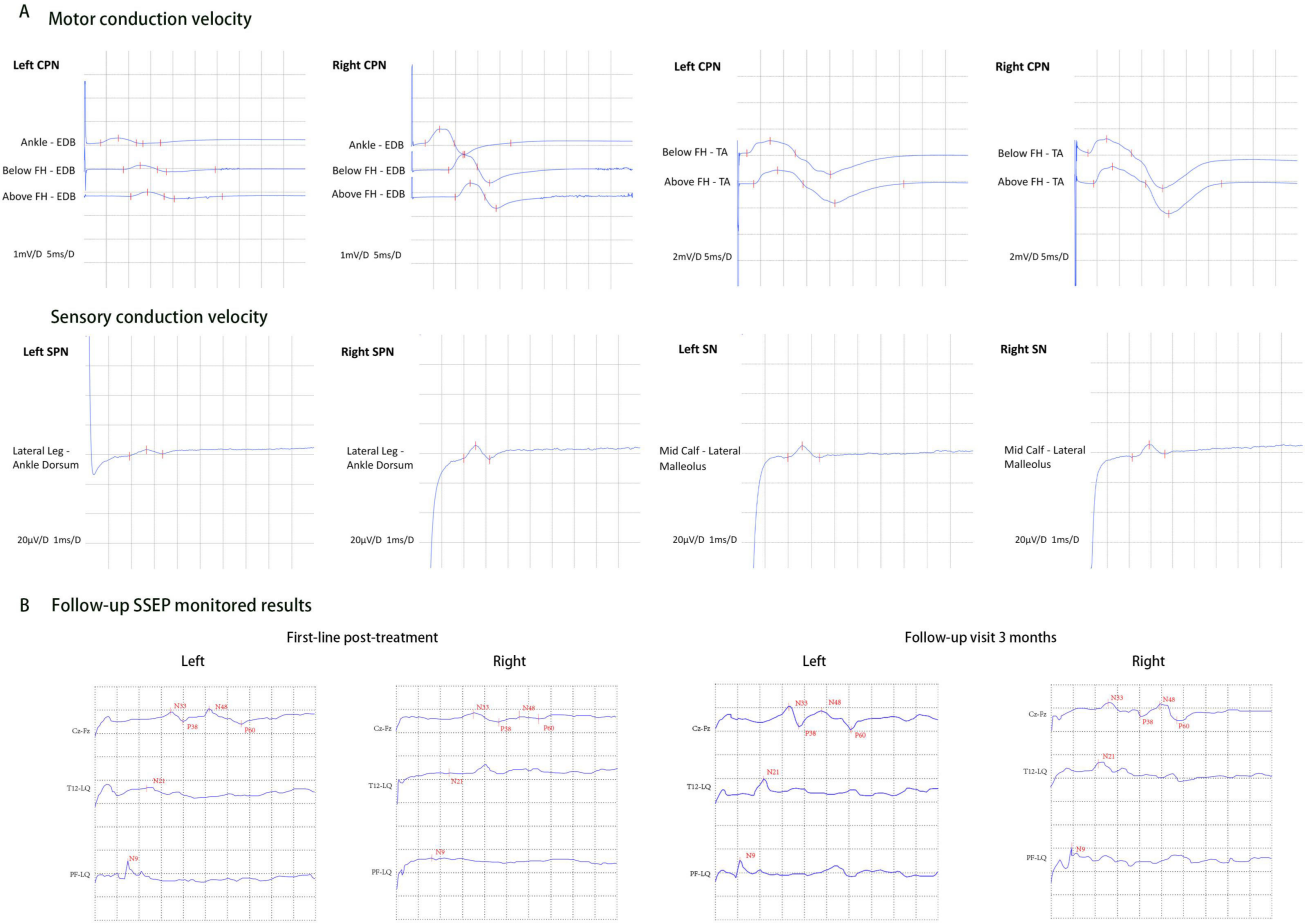
Results of electrophysiological examination. **(A)** The electrophysiological studies demonstrated that the compound muscle action potential amplitudes of the CPN were significant decreased bilaterally, as well as the sensory nerve action potential amplitude of the left SPN compared to the right. The results revealed evidence of asymmetric, multifocal axonal peripheral neuropathy. **(B)** SSEP monitoring at the 3-month follow-up demonstrated significant improvement in nerve conduction parameters, as shown by 26-61% reductions in N9 and N21 peak latencies and amplitude normalization. These electrophysiological findings suggest axonal regeneration and remyelination in previously compromised neural pathways. CPN, common peroneal nerve; SPN, superficial peroneal nerve; SN, sural nerve; EDB, extensor digitorum brevis; FH, flexor hallucis; TA, tibialis anterior; SSEP, somatosensory-evoked potentials.

After 8 days of high-dose intravenous methylprednisolone (IVMP) with no significant clinical improvement, combination therapy with intravenous immunoglobulin (IVIG) was initiated at a dose of 0.4 g/kg/day for 5 days, as the patient initially declined plasma exchange (PE) due to concerns about potential risks. The patient exhibited partial recovery of sensory deficits, with the sensory level from T2 descending to T8, although there was no recovery in motor function. A follow-up MRI revealed longitudinally extensive spinal cord swelling from C6-T10, with segmental reduction in edema compared to prior imaging. Gadolinium-enhanced MRI showed no significant enhancement of the spinal lesions, but the formation of syringomyelia at C7-T2 was seen (**Figures 1K, L**). Electrophysiological studies demonstrated that the compound muscle action potential amplitudes of the common peroneal nerves were decreased bilaterally, as well as the sensory nerve action potential amplitude of the left superficial peroneal nerve compared to the right. The results revealed evidence of asymmetric, multifocal axonal peripheral neuropathy (**Table 1**; **Figure 3A**). Visual evoked potential showed a significantly prolonged P100 latency on the right side, consistent with previous right optic neuritis.

**Table 1:** Nerve conduction study results in the lower limbs.

Nerve	Lat (ms) L/R	Amp (mV) L/R	CV (m/s) L/R	Reference values
Motor nerve conduction tests				
Tibial nerve	3.00/2.76	6.0/5.1	Not tested	Lat <4.5 ms; Amp >4 mV; CV >40 m/s
Common peroneal nerve (EDB)	3.71/3.21	0.47/2.1	53.7/52.7	Lat <6.5 ms; Amp >2 mV; CV >42 m/s
Common peroneal nerve (TA)	2.07/2.98	2.6/3.8	61.6/63.0	Lat <4.5 ms; Amp >5 mV; CV >50 m/s
Sensory nerve conduction tests				
Tibial nerve	13.39/13.73	2.2/3.2	44.2/40.2	Lat <14 ms; Amp >3 µV; CV >40 m/s
Superficial peroneal nerve	1.92/2.00	3.0/9.4	54.7/57.5	Lat <4.0 ms; Amp >6 µV; CV >40 m/s
Sural nerve	1.99/2.15	7.9/6.2	57.8/58.1	Lat <4.4 ms; Amp >6 µV; CV >40 m/s

Reference values were based on the electrophysiology laboratory standards of our institution for adults aged 18–60 years.

L, left; R, right; Lat, latency; Amp, amplitude; CV, conduction velocity; EDB, extensor digitorum brevis; TA, tibialis anterior.

The patient’s symptoms modestly resolved, with sensation restored caudally to the T12 dermatome. However, both lower limbs remained paralyzed with grade 0 muscle strength and she experienced episodes of lower limb myoclonus. Repeat CSF analysis showed an elevation in pressure to 190 mmH_2_O, and white cell count decreased to 5 × 10^6^/L, while protein slightly rose to 0.58 g/L. Glucose and chlorine were within normal ranges. The CSF IgG concentration had decreased to 37.7 mg/L, while the lgG index was 0.28. OCB analysis demonstrated a type IV pattern, indicating systemic immune activation and passive antibody transfer across a compromised blood-brain barrier (BBB). Repeat antibody testing yielded positive AQP4-Abs in serum at a titer of 1:100, with a decrease in CSF titer from 1:32 to 1:10. In contrast, AGO-Abs titers showed a marked increase in serum from 1:30 to 1:320 and remained detectable in CSF at a titer of 1:10 (**Figure 2A**).

Due to an insufficient response to IVMP and IVIG, therapeutic PE was initiated four weeks after IVIG therapy, at 1.5 plasma volume per session, every other day for a total of 5 times. During the course of PE, the patient reported some gradual restoration of voluntary toe movements, along with progressive improvement in sensory abnormalities. By the end of the PE treatment, she was able to lift her lower limbs off the bed for less than 5s. Neurological examination revealed proximal muscle strength in the bilateral lower limbs graded at 3, while distal strength was grade 1-2, with normal superficial sensation. Concurrently, maintenance immunosuppression therapy was initiated with the addition of mycophenolate mofetil (MMF) to a target of 750 mg twice daily. A repeat lumbar puncture found that intracranial pressure had decreased back to within normal ranges at 120 mmH_2_O. CSF white blood cells had elevated to 18 × 10^6^/L, predominantly polymorphonuclear cells (72.0%). CSF protein slightly decreased to 0.55 g/L, while glucose and chloride remained normal. Follow-up antibody testing showed a marked decline in AGO-Abs titers and CSF AQP4-Abs titers, while serum AQP4-Abs titers increased significantly to 1:320 (**Figure 2A**). Spinal MRI demonstrated resolution of the longitudinal spinal cord edema, with residual multifocal patchy hyperintensities on T2 imaging extending from levels T1 to T10. However, there was mild enlargement of the syringomyelia compared to prior imaging, now extending from C6 to T4 levels (**Figures 1M–O**). Somatosensory-evoked potentials (SSEP) testing revealed significant peripheral nerve injury characterized by prolonged latency and reduced amplitude (**Figure 3B**).

To achieve B-cell depletion and reduce risk of relapse, rituximab (RTX) was administered in two doses totaling 600 mg, based on a standard dose of 375 mg/m^2^. An initial 100 mg infusion was followed by a second dose of 500 mg three days later. The patient regained autonomous control over urination after RTX therapy. She reported no adverse effects such as joint or muscle pain from the treatment. At discharge, the patient’s condition had improved significantly, although she still required the use of a wheelchair, and scored 8.0 on EDSS.

**Figure 4:**
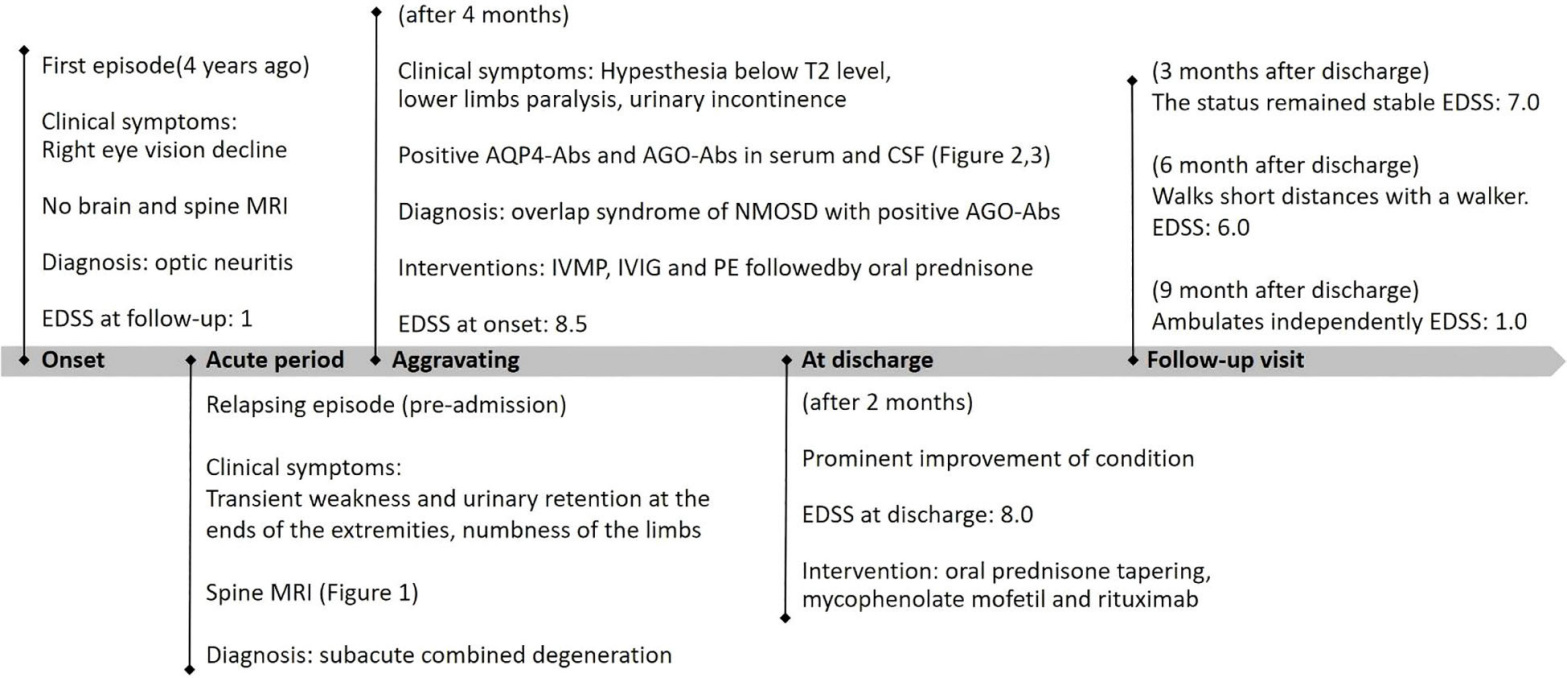
Timeline of the patient, with relevant episode and intervention data. MRI, magnetic resonance imaging; EDSS, expanded disability status scale; AQP4-Abs, aquaporin-4 antibodies; AGO-Abs, argonaute antibodies; CSF, cerebrospinal fluid; NMOSD, neuromyelitis optica spectrum disorder; IVMP, intravenous methylprednisolone; IVIG, intravenous immunoglobulin; PE, plasma exchange.

During follow-up 3 months post-discharge, her lower limbs strength had improved and she was able to stand with assistance, although she was still unable to ambulate (EDSS 7.0). Follow-up SSEP testing demonstrated shortened N9 and N21 latencies, along with restoration of wave amplitudes, indicating functional recovery of previously injured nerve fibers. At 9-month follow-up, the patient was able to walk independently, and had improved to an EDSS score of 1. Repeat antibody testing revealed persistently high serum antibody titers, while CSF titers remained low (**Figure 2A**). Neurological improvement was consistent with the stable findings on follow-up cervical MRI. Oral immunotherapy was continued without dose modification. The patient remains under regular clinical follow-up. A detailed timeline summarizing key clinical events, interventions, and corresponding data is provided in **Figure 4**.

## 3 Discussion

NMOSD is a rare autoimmune disease of the central nervous system characterized by antibody-mediated astrocytopathy, most commonly associated with AQP4-Abs (9). Together with myelin oligodendrocyte glycoprotein antibody-associated phenotypes, NMOSD is now considered a heterogeneous disease spectrum (10). The presence of AQP4-Abs is strongly associated with severe, relapsing neurological damage (11). However, a subset of initially AQP4-seronegative patients may exhibit atypical clinical features, remain asymptomatic, or follow a monophasic disease course. Notably, approximately 29.2% of these patients may later convert to a seropositive status, with a mean time to seroconversion exceeding 37.9 months (12).

The co-occurrence of NMOSD and AGO-Abs is extremely rare, with limited data available of a distinct overlap phenotype. Vitamin B12 deficiency initially introduced diagnostic uncertainty in our patient. The subsequent development of longitudinally extensive transverse myelitis and confirmation of AQP4-Abs positivity supported the diagnosis of NMOSD as the primary disease process rather than classical SCD. AGO-Abs have been predominantly identified in patients with sensory neuronopathy (SNN), limbic encephalitis, diencephalic or cerebellar syndromes, and polyneuropathies, with Sjögren’s syndrome frequently also occurring in SNN cases (13, 14). A prospective study systematically screened patients with suspected NMOSD and identified AGO-Abs in 7 of 104 patients (6.7%), including five individuals who were also AQP4-Ab positive (13). Notably, transverse myelitis was the predominant clinical manifestation, and one case presented with concomitant polyradiculopathy. In addition, a recently reported case by Liu et al. described simultaneous detection of AQP4-Abs and AGO-Abs in a patient presenting with relapsing brainstem syndrome and longitudinally extensive transverse myelitis, further supporting the concept of an NMOSD-AGO overlap autoimmune features with heterogeneous clinical phenotypes (15). These findings suggest that the presence of AGO-Abs may define a subgroup of NMOSD patients characterized by prominent spinal cord involvement and immune complexity. Our patient who exhibited asymmetric axonal sensorimotor polyneuropathy alongside longitudinally extensive transverse myelitis may reflect broader neuro-immunological involvement across central and peripheral nervous systems. While AQP4-Abs are known to cause immune responses and complement activation directed at astrocytes, the pathogenic role of AGO-Abs in NMOSD remains unclear. The presence of both antibodies may reflect heightened immune dysregulation or a synergistic pathogenic mechanism. These findings suggest that in patients with suspected sensory nerve involvement, comprehensive diagnostic evaluation—including CSF analysis and serologic testing for AGO-Abs—may be warranted. Further large-scale, prospective cohort studies are needed to characterize the clinical spectrum of AGO-Abs-positive NMOSD and determine whether AGO-Abs serve as specific biomarkers for extensive transverse myelitis or diencephalic syndromes within the NMOSD spectrum.

Antibody coexistence in systemic autoimmune diseases is rarely reported, although it appears to be more common in NMOSD (16). In our case, although the patient was seropositive for multiple auto-antibodies, including ANA, AMA, AMA-M2, and anti-intrinsic factor antibodies, there were no clinical features suggestive of underlying connective tissue diseases or other rheumatologic syndromes. Furthermore, the diagnostic criteria for these autoimmune diseases were not met. In one study, 80.77% of NMOSD patients with persistently low-titer ANA positivity showed no direct pathogenic relevance to the disease, but this finding may reflect an underlying systemic immune dysregulation rather than specific pathogenic relevance (17). AMA, AMA-M2, and anti-intrinsic factor antibodies are typically associated with primary biliary cholangitis, autoimmune gastritis, or pernicious anemia; however, our patient did not exhibit clinical or laboratory evidence of these conditions. These low-titer antibodies underscore the complexity of immune dysregulation in NMOSD, particularly during pregnancy and the postpartum period (1).

Although OCB analysis and IgG index are not formally required for the diagnosis of NMOSD under current diagnostic criteria, they may offer further insight into immune dynamics. Initially, our patient exhibited type I OCB, which later evolved to type IV following treatment with IVMP and IVIG. This may indicate persistent B-cell activation and BBB disruption, potentially reflecting suboptimal efficacy of immunosuppressive treatment efficacy (18). Nonetheless, the diagnostic value of OCB and the IgG index in NMOSD remains limited, particularly in patients with moderate to severe BBB dysfunction (19). Hence, their routine use should be interpreted with caution in clinical practice and in conjunction with other indicators.

Early neurological improvement following vitamin supplementation initially supported a diagnosis of subacute combined degeneration, given the mildly reduced vitamin B12 level and positive anti–intrinsic factor antibodies. However, NMOSD typically does not to respond to vitamin replacement therapy, suggesting that the transient clinical improvement more likely reflected partial correction of metabolic dysfunction or concomitant peripheral nervous system involvement rather than modification of the underlying inflammatory disease. Electrophysiological studies demonstrated asymmetric axonal sensorimotor polyneuropathy rather than classical SCD-related posterior column dysfunction. Considering reported associations between AGO-Abs and immune-mediated peripheral neuropathies, coexisting autoimmune peripheral nerve involvement together with mild vitamin B12 deficiency may explain the temporary treatment response and early diagnostic confusion preceding recognition of NMOSD.

In severe attacks of AQP4-positive NMOSD, particularly in patients presenting with complete paraplegia, current evidence supports early initiation of PE preferably in combination with IVMP, as an effective escalation strategy (20). Multiple studies have demonstrated that earlier implementation of PE is associated with improved neurological outcomes, especially in patients with insufficient response to IVMP monotherapy or those positive for AQP4-Abs (21). Although the European Federation of Neurological Societies recommends PE as a second-line treatment in the acute phase, a systematic review has demonstrated that IVIG is generally less effective than PE in improving EDSS scores (22). In our case, IVMP and subsequent IVIG therapy resulted in limited clinical improvement, whereas PE led to marked neurological recovery. Antibody monitoring demonstrated a more pronounced decline in both serum and CSF antibody titers following PE compared to prior therapies, although serum AQP4-Abs titers remained elevated. Previous studies have reported an association between higher AQP4-Abs titers and more severe disease activity and relapse risk (23). Notably, SNN associated with AGO-Abs positivity has been reported to respond favorably to IVIG therapy (14). However, treatment strategies for NMOSD patients with coexisting AGO-Abs remain poorly defined and are largely extrapolated from classical NMOSD cohorts (13). In the present case, the limited response to IVIG contrasts with prior observations in AGO-Abs-positive SSN, suggesting that treatment response was more likely driven by established NMOSD treatment principles rather than AGO-Abs status alone. The substantial clinical improvement observed after PE may therefore primarily reflect its known efficacy in treating severe AQP4-positive NMOSD attacks. However, the concomitant reduction in AGO-Abs titers raises the possibility that antibody dynamics contribute to treatment response.

Moreover, the sequential and alternating therapy of PE and IVIG, known as the “zipper method”, have been proposed as rescue approaches in severe or refractory autoimmune neurological disorders, particularly in antibody-mediated diseases. This approach has been shown to improve clinical outcomes and reduce the proportion of non-responders (24). In the present case, however, both antibodies remained detectable in serum at varying titers despite neurological recovery. This dissociation between antibody levels and clinical status suggests that antibody dynamics alone may not fully explain treatment response, a phenomenon previously reported in NMOSD, where clinical recovery does not always parallel serological changes. Although the concomitant reduction in antibody titers raises the possibility of an immunological contribution to treatment response, this association remains speculative and requires confirmation in larger cohorts. Notably, AGO-Abs positivity does not necessarily predict poor recovery but may instead reflect an expanded autoimmune spectrum, supporting the importance of comprehensive antibody screening in patients with atypical or overlapping phenotypes. Nevertheless, evidence supporting optimal escalation strategies in NMOSD patients with overlapping autoimmune antibody profiles remains limited, and randomized controlled studies addressing combination or sequential immunotherapies are still needed.

To prevent relapse, early initiation of immunotherapy is critical. B-cell depletion with RTX is a cornerstone of maintenance treatment in AQP4-Abs-positive NMOSD. In our patient, the serum CD19+ B-cell proportion remained elevated (15.22%) following acute-phase treatment, despite IL-6 levels being within the normal range. Based on this indicator, we initiated RTX therapy, along with prescribed oral MMF and low-dose glucocorticoids. Indeed, it is recommended that MMF is combined with oral glucocorticoids for at least the first 3 months of therapy (25). Although combination therapies (i.e., monoclonal antibodies plus immunosuppressants) may offer benefits for patients with multiple autoantibodies or overlap syndrome, there is a dearth of robust evidence comparing monotherapy versus combination therapy (26). Furthermore, due to the limited availability of prospective data, the optimal duration of combined immunosuppressive therapy and the strategy for discontinuing maintenance immunosuppressants in patients with multiple autoantibodies or during pregnancy remains unclear.

While the EDSS remains the standard for assessing neurological function, complementary tools—including SSEP, MRI, and optical coherence tomography—enhance the ability of clinicians to track progression and neuroaxonal atrophy (27–29). Although antibody titers are not routinely recommended for monitoring, their changes may be relevant to clinical activity in select cases in which undergoing B-cell depletion therapy or IL-6 receptor blockade may transiently produce negative AQP4-Abs results (30). In complex cases with overlapping autoimmune conditions, the value of standardized comprehensive multimodal assessment—incorporating clinical, serological, imaging, and electrophysiological evaluations—in guiding subsequent treatment decisions and prognostic assessment requires further validation to facilitate integrated disease management throughout the entire clinical course.

## 4 Summary

This case illustrates AQP4-positive NMOSD with concomitant anti-AGO-Abs positivity and peripheral nervous system involvement, highlighting the expanding immunological spectrum of NMOSD and the potential for diagnostic confusion with peripheral neuropathic disorders. Broad-spectrum antibody screening is essential in individuals with limited, or atypical neuroimmunological presentations. Plasma exchange appeared clinically beneficial in the acute phase, while combined immunotherapy supported sustained recovery. Persistent antibody positivity despite clinical recovery suggests that serological changes do not necessarily parallel disease activity. Further studies are required to clarify the clinical significance of AGO-Abs and to optimize management strategies in patients with complex autoimmune antibody profiles.

## Data Availability

The original contributions presented in the study are included in the article/**Supplementary Material**. Further inquiries can be directed to the corresponding author/s.
